# Simplifying Wheat Quality Assessment: Using Near-Infrared Spectroscopy and Analysis of Variance Simultaneous Component Analysis to Study Regional and Annual Effects

**DOI:** 10.1021/acsmeasuresciau.4c00044

**Published:** 2024-10-04

**Authors:** Stephan Freitag, Maximilian Anlanger, Maximilian Lippl, Klemens Mechtler, Elisabeth Reiter, Heinrich Grausgruber, Rudolf Krska

**Affiliations:** † Department of Agrobiotechnology, IFA-Tulln, Institute of Bioanalytics and Agro-Metabolomics, BOKU University, Konrad-Lorenz-Str. 20, 3430 Tulln an der Donau, Austria; ‡ Institute for Animal Nutrition and Feed, Austrian Agency for Health and Food Safety GmbH, Spargelfeldstr. 192, 1220 Vienna, Austria; § Institute for Sustainable Plant Production, Austrian Agency for Health and Food Safety GmbH, Spargelfeldstr. 192, 1220 Vienna, Austria; ∥ Department of Crop Sciences, Institute of Plant Breeding, BOKU University, Konrad-Lorenz-Str. 24, 3430 Tulln an der Donau, Austria; ⊥ FFoQSI GmbH−Austrian Competence Centre for Feed and Food Quality, Safety and Innovation, Technopark 1C, 3430 Tulln an der Donau, Austria; # Institute for Global Food Security, School of Biological Sciences, Queens University Belfast, University Road, Belfast, Northern Ireland BT7 1NN, U.K.

**Keywords:** near-infrared spectroscopy, wheat, chemometrics, ASCA, green analytical chemistry

## Abstract

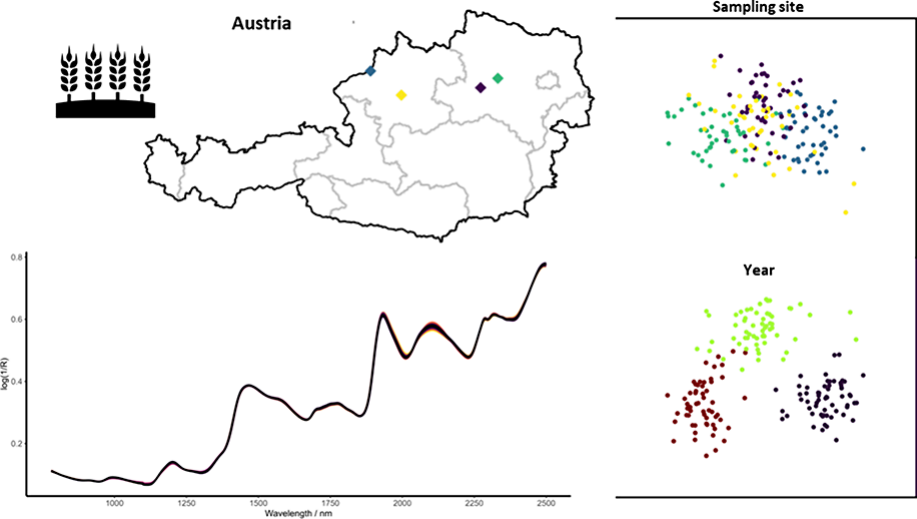

Assessing the quality of wheat, one of humanity’s most important crops, in a straightforward manner, is essential. In this study, analysis of variance (ANOVA) simultaneous component analysis (ASCA) paired with near-infrared spectroscopy (NIRS) was used as an easy-to-implement and environmentally friendly tool for this purpose. The capabilities of combining NIRS with ASCA were demonstrated by studying the effects of sampling site and year on the quality of 180 Austrian wheat samples across four sites over 3 years. It was found that the year, sample site, and their combination significantly (*p* < 0.001) affect the NIR spectra of wheat. NIR spectral preprocessing tools, usually employed in chemometric workflows, notably influence the results obtained by ASCA, particularly in terms of the variance attributed to annual and regional effects. The influence of the year was identified as the dominant factor, followed by region and the combined effect of year and sampling site. Interpretation of the loading plots obtained by ASCA demonstrates that wheat components such as proteins, carbohydrates, moisture, or fat contribute to annual and regional differences. Additionally, the protein, starch, moisture, fat, fiber, and ash contents of wheat samples obtained using a NIR-based calibration were found to be significantly influenced by year, sampling site, or their combination using ANOVA. This study shows that the combination of ASCA with NIRS simplifies NIR-based quality assessment of wheat without the need for time- and chemical-consuming calibration development.

## Introduction

Wheat (*Triticum L.*) is one of the most important agricultural crops that feeds humanity. In the year 2023, 795.7 million tons of wheat were produced worldwide, of which 65% were used as food and 19% as feed.^1^ Due to the increasing global population in a changing climate, there is a growing demand for cost-efficient, rapid, and environmentally friendly tools to be used in the food supply chain. Determining the protein, moisture, and carbohydrate content of wheat, among other parameters, has been routinely performed with near-infrared spectroscopy (NIRS) since the 1970s.^2^ Following this early implementation, a wide variety of applications using NIRS for wheat quality assessment have been reported.^3^ The nondestructive character of NIRS, requires minimal sample handling procedures and the consequently cost-effectiveness and greenness of the technique have led to its widespread use not only in the agricultural sector.^4^ However, these advantages of NIRS are only exploitable when a calibration for the parameter of interest such as protein or moisture is available.^5^ Developing these calibrations usually based on multivariate statistics is a complex procedure and requires costly, complicated and nongreen reference analysis, being one of the major disadvantages of NIRS.

In the early 2000s, analysis of variance simultaneous component analysis (ASCA) was introduced to be used with so-called designed metabolomics data sets.^6^ ASCA combines analysis of variance (ANOVA) with component analysis.^7^ Thus, the technique allows the study of the significance of effects on multivariate data in a straightforward manner besides decomposing the data along the effects of interest. Since the introduction of ASCA, the technique has been used not only in omics-based methods but also with spectroscopic data.^7^ ASCA has been paired with NIRS to study the effects of freezing on donkey milk,^8^ drying on mango fruit,^9^ staling of bread,^10^ for pasta characterization,^11^ manipulation of honey,^12^ and wheat roasting.^13^ The implementation of ASCA only requires an experimental design that takes the effect to be tested into account. Recently, the concept of ASCA has been further expanded to allow the analysis of nonbalanced experimental designs (ASCA+).^14^

In chemometric workflows spectra preprocessing is a crucial step when using NIR data.^5,15,16^ Often, a wide range of spectra preprocessing methods and their combination is explored in the process of calibration or model development and chosen empirically based on the performance parameters of the final calibration. Multiplicative scatter correction (MSC), standard normal variate (SNV), or derivatives paired with Stavisky-Golay smoothing are widely used spectra-preprocessing techniques.^15,16^ While MSC is a model-based approach using a reference spectrum,^17^ during SNV,^18^ the standard normal variate at each individual wavelength throughout the NIR spectrum is calculated. Both MSC and SNV aim to remove nonlinearities such as scatter effects. The concept of MSC has been further extended (EMSC) to improve the separation of chemical and physical information during spectral processing.^19^ Derivatives of the NIR spectra remove baseline drifts and enhance the obtained spectral information, often at the cost of also enhancing noise.^16^ It has been demonstrated that ASCA is sensitive to data preprocessing steps,^7^ such as scaling or centering.

The aim of this study was to explore the usability of ASCA combined with NIRS to study regional and annual effects on wheat. We also investigated the influence of spectra preprocessing on ASCA results. Our findings demonstrate that wheat composition is significantly (*p* < 0.001) influenced by year, sampling site, and their interaction, with preprocessing notably affecting ASCA outcomes. Moreover, ASCA results closely align with those from a routine NIR calibration, highlighting the reliability of this approach for assessing wheat quality.

## Materials and Methods

### Sample Collection

**Figure 1:**
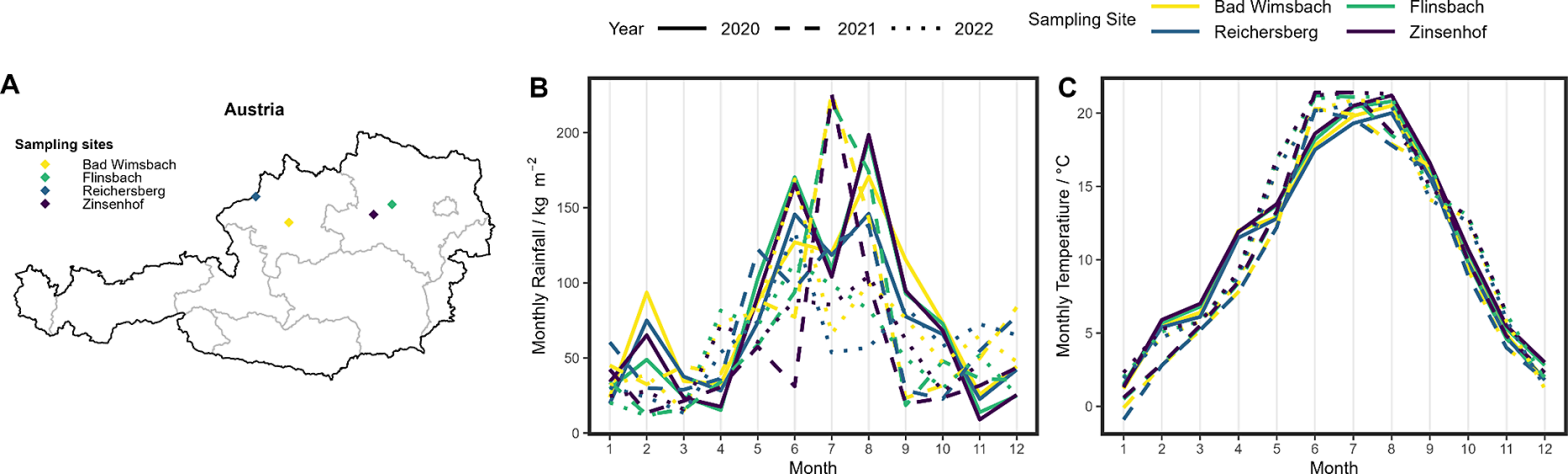
(A) Map of Austria indicating the sampling sites. (B) Averaged monthly rainfall for the sampling sites. (C) Averaged monthly temperature for the sampling sites.

Wheat samples were collected during the value for cultivation and use testing program of the Austrian Agency for Health and Food Safety (AGES).^20^ A total of 180 wheat samples were selected for 2020, 2021, and 2022. Samples were obtained from four different sampling sites, i.e., Bad Wimsbach, Flinsbach, Reichersberg, and Zinsenhof, per site, and year 15 samples were collected. In Figure 1 a map of Austria indicating the sampling sites is shown, GPS data of the locations can be found in the Supporting Information. The weather data for each sampling site was sourced from the Spartacus data set^21^ and can be seen for the three sampling years in Figure 1B,C. Wheat variety trials were performed in three or four replicates using a rectangular lattice design at each site. Single equally sized subsamples were taken from each of the four replicates and bulked (approximately 10 kg), resulting in one sample per variety, region, and year. A reduced composite sample ranging from 2 to 8 kg per variety was transferred to the laboratory for grinding. The wheat samples were ground to a particle size <0.8 mm with a Chopin grinder (KPM Analytics, Villeneuve-la-Garenne, France). Subsequently, 200 g of each sample was collected for NIRS analysis.

### Near-Infrared Spectroscopy

**Figure 2:**
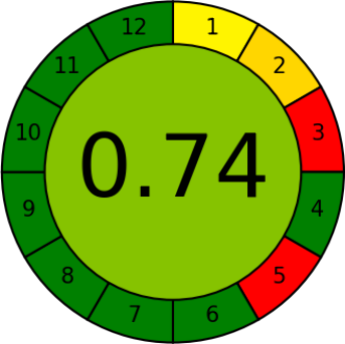
AGREE score obtained for the NIR method based on the 12 principles of green analytical chemistry. 1: What sampling procedure is needed; 2: The amount of sample needed; 3: Placement of the instrument; 4: Required sample handling steps; 5: Degree of automatization of sample handling; 6: Usage of derivatization; 7: The amount of waste generated; 8: The number of analytes determined per run and the sample throughput per hour; 9: energy requirements; 10: type of reagents; 11: toxicity of reagents; 12: operator safety.

NIR spectra were recorded using a Foss DS 2500 (Foss, Hilleroed, Denmark) benchtop spectrometer (400–2499.5 nm, 0.5 nm spectral resolution). The small sampling cup and the cereal calibration were used to record the spectra and to obtain the protein, moisture, fat, fiber, ash, and starch contents of the wheat samples. Spectra were exported as an .NIR batch file and converted into a .csv file using SpectraGryph (Spectroscopy Ninja, Oberstdorf, Germany). The greenness of the used NIR-method was evaluated using the evaluation tool AGREE,^22^ yielding a greenness score of 0.74 (see Figure 2).

In Figure 2, the graphical representation of the greenness score obtained by the AGREE metric can be seen. The score is based on the following input: off-line analysis (principle 1), using 5 g of sample (principle 2), with the instrument placed off-line in a laboratory (principle 3). The samples were milled (principle 4), which was done manually (principle 5). No reagents were used, resulting in no waste (principles 6, 7, 10, 11, and 12). Thirty samples were analyzed per hour, and the number of analytes was six (protein, moisture, starch, fiber, ash, and fat; principle 8). The energy requirements were assumed to be the same as for a Fourier transform infrared spectrometer, as suggested by the application (principle 9).

### Data Analysis

Data analysis was performed using the statistical computing software R4.2.2 in the development environment Rstudio (Posit, PBC, Boston, Massachusetts). Spectra preprocessing was performed in the packages prospectr and emsc.^23,24^ For principal components analysis (PCA), the package mdatools was used.^25^ ASCA was performed in the R package limpca.^26^ For plotting, ggplot2 was used.^27^ After performing PCA on the raw NIR spectra, Hotelling and orthogonal distances were used to identify outliers.^25^ In total, three outliers were found. Outliers are indicated in the Supporting Information. After outlier removal, only the NIR region (780 to 2499.5 nm) was used for further data analysis. The Savitzky–Golay gap-segment derivatives were calculated using a segment and gap size of 21. For EMSC, a fourth-degree polynomial was employed. For both MSC and EMSC the average of all spectra served as the reference spectrum after outlier removal. Bootstrapping using 1000 repetitions was used to test the significance of the effects on the spectral data during ASCA.^26^ Further details on how the amount of variance attributed by ACSA to the certain effects are calculated can be found in Thiel el al.^14^ The effect of the region and year on sample composition (protein, moisture, fat, fiber, ash and starch) obtained by the built-in calibration of the NIR spectrometer was analyzed by ANOVA using the basic aov function of R.

## Results and Discussion

**Figure 3:**
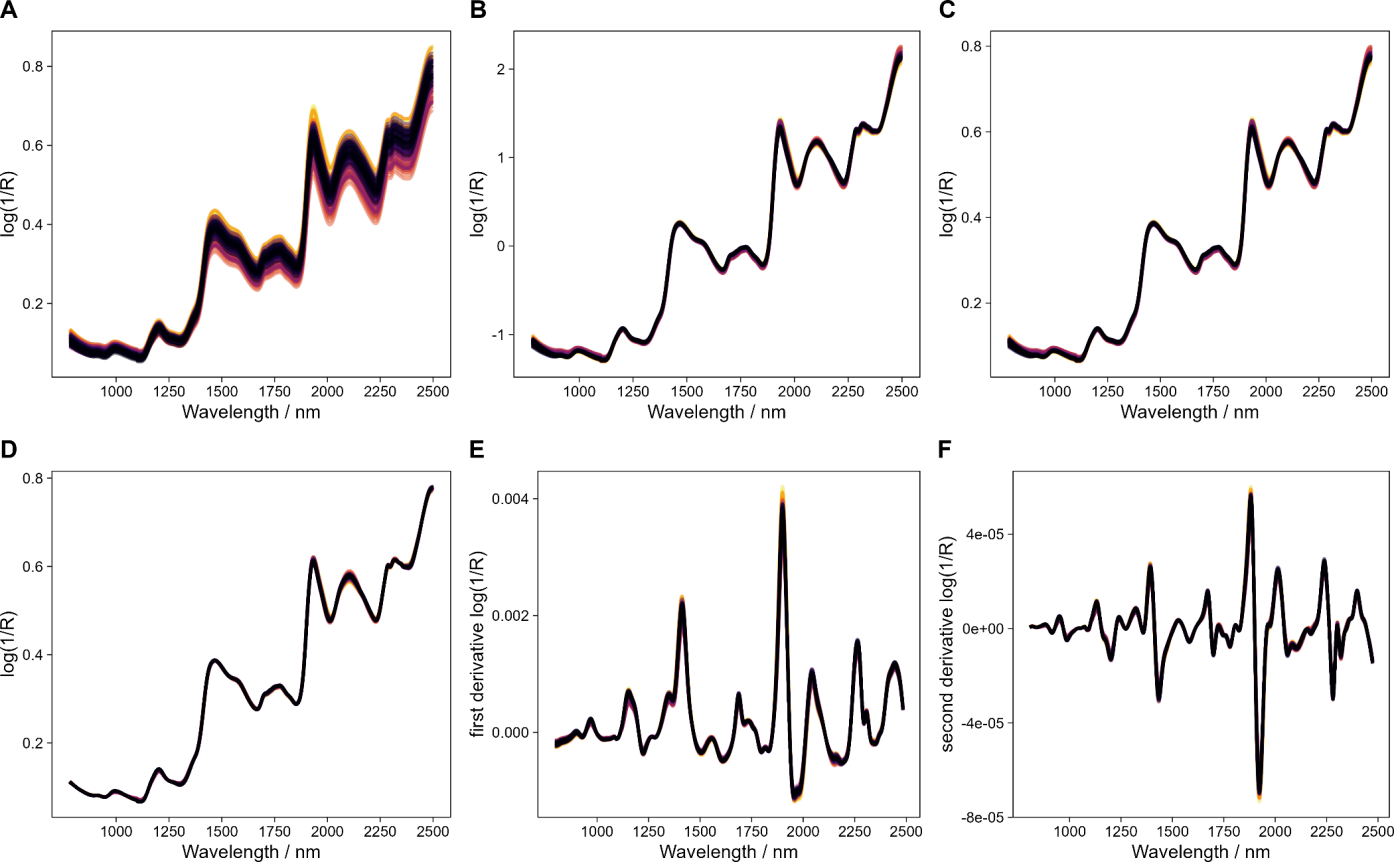
Raw and processed near-infrared spectra of milled wheat grain. Raw spectra (A), spectra processed using SNV (B), MSC (C), EMSC (D), the first derivative (E), and the second derivative (F).

After recording the NIR spectra (780–2499.5 nm), the spectra processing methods SNV, MSC, EMSC, and derivatives were employed to study their influence on the results obtained by ASCA. In Figure 3, the raw and processed NIR spectra of the wheat samples are shown. When comparing the SNV, MSC, and EMSC processed spectra (Figure 3B–D), only subtle differences were observed between the spectra processing methods. The most prominent difference appears to be the scaling of the *y*-axis when using SNV. Calculating the first and second derivatives (Figure 3E,F) removes the baseline drift and enhances the spectral information. However, by simply plotting the differently processed spectra, choosing the most suitable processing method is challenging. Therefore, the differently processed spectra were subjected to ASCA.

**Figure 4:**
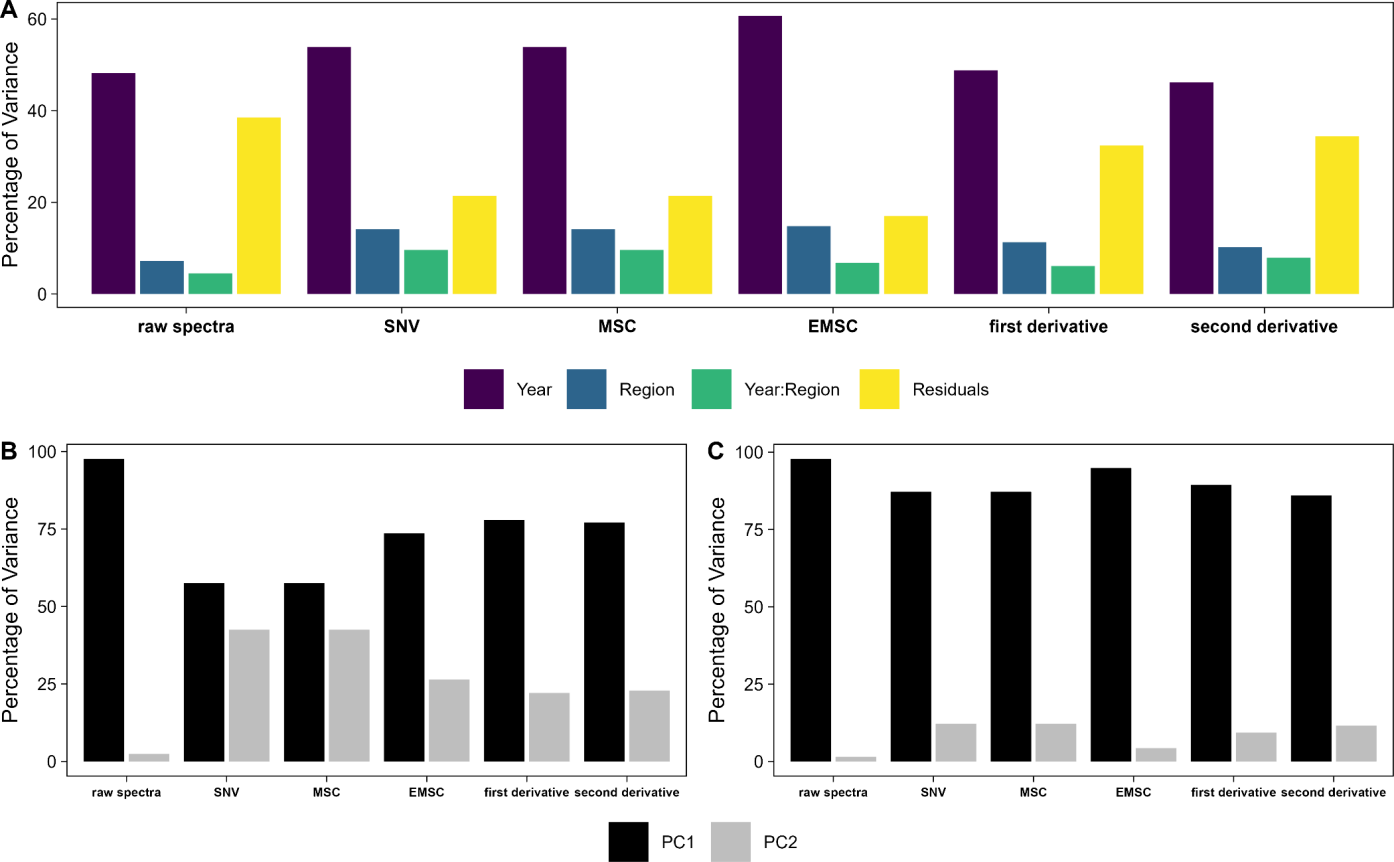
(A) Percentage of variance explained by analysis of variance simultaneous component analysis (ASCA) on the raw near-infrared spectra, standard normal variate (SNV) corrected, multiplicative scatter corrected (MSC), extended multiplicative scatter corrected (EMSC), and first and second derivatives of the spectra. Influence of the variance captured by the first two principal components (PCs) during ASCA decomposition and dimension reduction for the effect of the year (B) and sampling site (C).

As shown in Figure 4, spectral processing has a notable effect on the results obtained by ASCA. It was found that year, region and their interaction have a significant effect (*p* < 0.001) on the NIR spectra, regardless of which spectral processing method was employed. However, the amount of residual variance or variance linked to certain effects varied considerably (Figure 3A). The use of raw spectra resulted in a notable amount of the variance remaining unexplained by ASCA (38.5%). Interestingly, when calculating the first (32.4%) and second derivative (34.4%) of the spectra the unexplained variance remained high compared to SNV (21.5%), MSC (21.4%) and EMSC (17%). This can be linked to the increase in noise when calculating the derivatives of spectra. When employing EMSC, the residual variance dropped notable compared with the other spectral preprocessing methods. This is linked to the fact that EMSC is the spectral preprocessing method capable of removing most of the nonlinearities found in the NIR spectra. In general, the dominating effect causing the most variance within the NIR data set was the year, followed by the sampling site and a combination of year and site. The amount of variance linked to the year ranged from 60.7 to 46.2% depending on the spectra preprocessing method. For the sampling site a range from 7.2 to 14.8% was found. The year by region interaction contributed 4.5 to 7.9% of variance. The exact amount for each spectra preprocessing method can be found in the Supporting Information. The variance explained by the principal components (PCs) obtained from ASCA decomposition and dimension reduction is influenced by the spectra preprocessing method used, as depicted in Figure 4B,C. For raw spectra, the majority (>97%) of the variance after decomposition across years or regions is captured in PC1.

Analysis of the loadings of PC1 indicates that this variance primarily originates from scatter effects, as expected (see Supporting Information). When applying preprocessing methods, there is a noticeable decrease in the variance captured in PC1 and a notable increase in PC2, especially evident when considering the effect of the year, compared to the use of unprocessed spectra. The highest variance is found in PC1 when using the first and second derivative of the spectra, followed by EMSC (see Figure 4B). A similar trend is observed for the analysis by sample site, where EMSC captures the most variance in PC1, followed by the first and second derivative of the NIR spectra (see Figure 4C).

**Figure 5:**
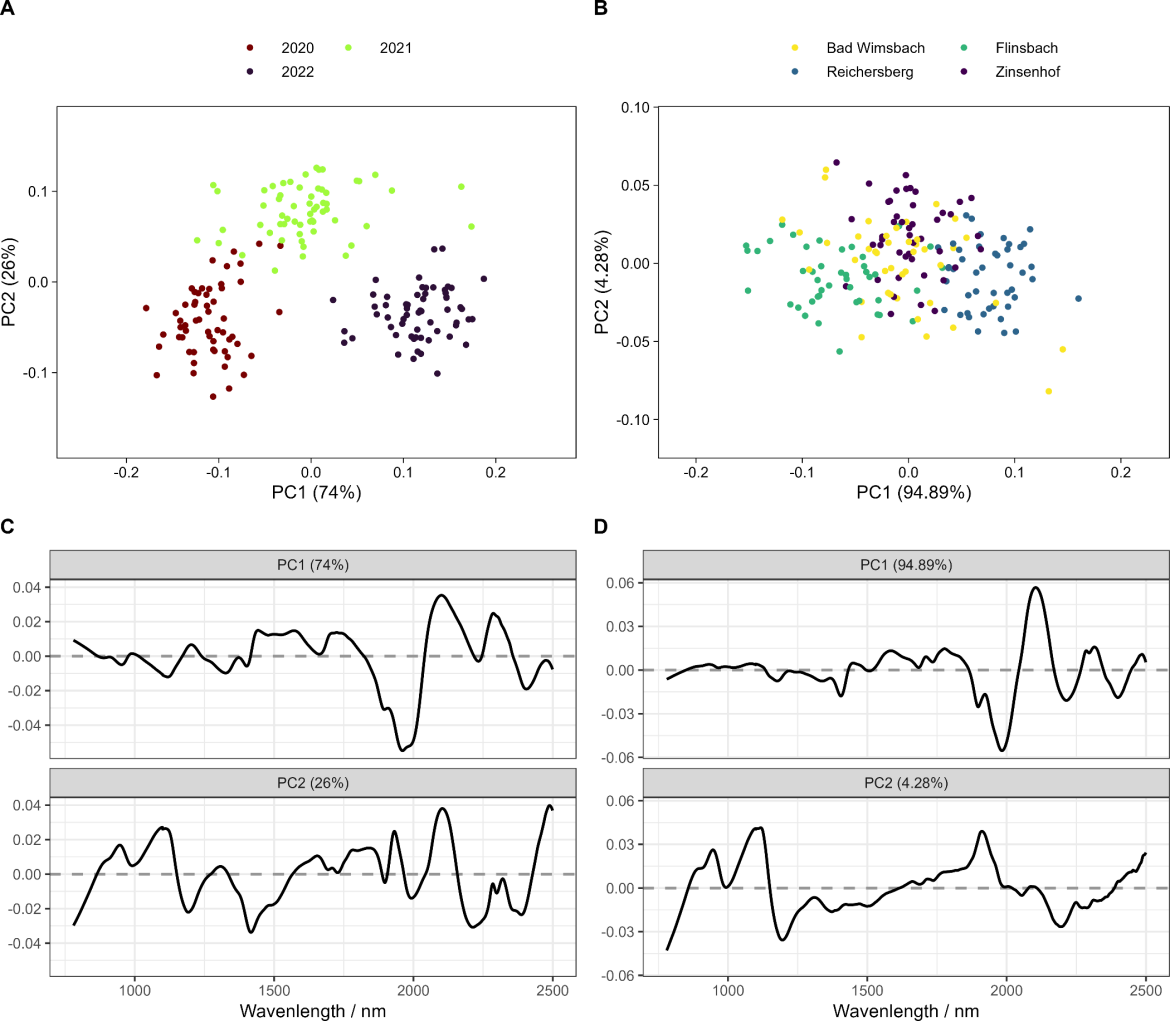
Score and loadings plots obtained by analysis of variance simultaneous component analysis (ASCA) using extended multiplicative scatter (EMSC) corrected near-infrared spectra. ASCA score plots for the effect of the year (A) or sampling site (B) as well as the corresponding loading plots for the effect of the year (C) or sampling site (D).

During ASCA decomposition of the data set, clear clusters linked to the year are formed in the score plots, the different regions are not as clearly separatable (see Figure 5). We hypothesize that the higher amount of variance attributed to the annual differences and the better-separated clusters for the different years found by ASCA are linked to larger annual weather differences affecting wheat growth compared to regional weather differences (see Figures 1B,C and 5). This is especially evident as some of the sampling sites are relatively close to each other, which also explains the overlap between them (see Figure 5B), as the environmental conditions were rather similar for these sampling sites within one year, but different between the years (see Figure 1B,C). This also aligns well with the distances between the sampling sites. Some sites are only 25 km apart (Zinsenhof/Flinsbach), while others are 140 km apart (Flinsbach/Reichersberg). Samples from the latter case are relatively separated (see Figure 5B). In Figure 5, results obtained after EMSC preprocessing are shown, as this combination led to the most variance explained by ASCA (see Figure 4). Score and loading plots for the other preprocessing methods paired with ASCA are shown in the Supporting Information.

The loadings linked to the annual or regional effect shown in Figure 5C,D can be linked to moisture (1440–1470 nm, 1920–1940 nm), protein (2148–2200 nm), fat (1725, 2310 nm), fiber (1206, 1450, 1664, 1820, 1940, 2000, 2242, 2300 nm), and starch (1063–1639, 1834–2175 nm) content of the samples.^3,5^ By performing band assignment on the ASCA loadings, it becomes clear that the regional and annual differences are linked to differences in protein, moisture, fat and carbohydrate content.

In addition to analyzing the obtained spectra, the calibration supplied by the NIR spectrometer for routinely analyzing cereal samples was used to predict the composition of the wheat samples. The *p*-values obtained by ANOVA to study the influence of region and year on the predicted values are shown in Table 1.

**Table 1:** *p*-Values Obtained by Analysis of Variance

parameter	year	region	year × region
protein	0.187	<0.001	<0.001
moisture	<0.001	<0.001	<0.001
fat	<0.001	<0.001	<0.001
fiber	<0.001	0.048	0.063
ash	<0.001	0.003	0.662
starch	<0.001	<0.001	<0.001

The findings obtained by ASCA are further underlined by the fact that when obtaining protein, moisture, fat, fiber, ash and starch content by an on-demand calibration embedded in the NIR spectrometer and subjecting the obtained values to ANOVA, a significant effect for either the year, region as well as their interaction are found. This shows that pairing ASCA with NIRS is a strong tool for studying influences on the data set by simply using spectra interpretation without the need for more complex calibrations or reference analysis. Furthermore, this ASCA-based approach might unravel effects that are not found with ANOVA on the sample composition obtained from a NIRS-based calibration. The NIR-based analytical method used in this study is inherently green, as only sampling and grinding are needed to record the spectra (see Figure 2). Current approaches for evaluating the greenness of chemometric-based analytical methods ignore the influence of the necessary reference analyses, to build NIR-based calibrations. Instead the chemometric method is compared to the reference method it should replace.^28^ However, chemometric approaches like ASCA, which do not require any reference analysis but only an experimental design or hypothesis, must be considered greener compared to the development of a classic calibration. To draw the conclusions with an NIR-based calibration as presented in Table 1, six different reference methods would be needed, all of which are expected to have a higher environmental impact than NIRS. However, the needed chemicals and the corresponding waste of these reference methods are usually not considered when evaluating the greenness of NIR-based methods as explained above.

Our findings on the observed effect of the sampling site and region are in good agreement with the literature. Previous research has shown that NIRS can be used to discriminate between wheat from different geographical regions and years.^29−33^ For discriminating wheat samples according to their origin, the chemometric tools linear discriminant analysis and/or partial least-squares discriminant analysis (PLS-DA) were used. Two studies used multiway ANOVA on the PCs obtained by PCA.^31,32^ In contrast to that studies ASCA can be used to study multiple effects and their interaction at once. Furthermore, the usage of ASCA, leads to a better interpretability of the results compared with a multiway ANOVA performed on PCs, as decomposition of the data set along those effects as well as dimension reduction is paired with ANOVA in ASCA. In recent studies, ASCA has been employed as a tool to assess the performance of different portable NIR spectrometers.^34,35^ Similar to this research, we also found a notable effect of spectral processing on the results obtained by ASCA. This underlines our statement that ASCA is a valuable tool for building chemometric models based on spectral data.

## Conclusions

We have shown that pairing ASCA with NIRS is a straightforward and environmentally friendly tool to study the influence of the sampling site and year on wheat quality. In contrast to other approaches, such as discriminant analysis, it allows to study multiple effects and their interaction simultaneously. During ANOVA on the protein, starch, moisture, ash, fat and fiber content of the wheat samples, either the year or sampling site, as well as the interaction of sampling site and year had a significant effect on all parameters. We linked the significant (*p* < 0.001) effect found by ASCA with absorbance features of protein, moister, carbohydrates and fat, by simply interpreting the obtained loading plots, thus by using ASCA the same conclusion can be drawn without the need for complex reference analysis. The choice of NIR spectral processing has a notable influence on the amount of variance attributed by ASCA to the different effects. This shows that ASCA is a valuable tool when evaluating different spectral preprocessing methods during chemometric work-flows. Follow-up research will leverage this advantage to investigate the possibility of establishing models for analyzing Austrian wheat for its authenticity.

## Supporting Information Available

NIR spectra, year and sampling site (XLSX)
The GPS location of the sampling sites the data as tables presented in Figure 4 and ASCA score and loadings plots obtained using the raw NIR spectra as well as SNV, MSC, first and second derivative treated spectra (PDF)

## References

[ref1] OECD-FAO. OECD-FAO Agricultural Outlook 2023–2032; OECD Publishing: Paris, 2023.

[ref2] McClure W. F. 204 years of near infrared technology: 1800–2003. J. Near Infrared Spectrosc. 2003, 11 (6), 487–518. 10.1255/jnirs.399.

[ref3] Du Z.; Tian W.; Tilley M.; Wang D.; Zhang G.; Li Y. Quantitative assessment of wheat quality using near-infrared spectroscopy: A comprehensive review. Compr. Rev. Food Sci. Food Saf. 2022, 21, 2956–3009. 10.1111/1541-4337.12958. 35478437

[ref4] Manley M. Near-infrared spectroscopy and hyperspectral imaging: non-destructive analysis of biological materials. Chem. Soc. Rev. 2014, 43 (24), 8200–8214. 10.1039/C4CS00062E. 25156745

[ref5] Williams P.; Manley M.; Antoniszyn J. Near infrared technology: getting the best out of light; African Sun Media, 2019.

[ref6] Smilde A. K.; Jansen J. J.; Hoefsloot H. C.; Lamers R.-J. A.; Van Der Greef J.; Timmerman M. E. ANOVA-simultaneous component analysis (ASCA): a new tool for analyzing designed metabolomics data. Bioinform. 2005, 21 (13), 3043–3048. 10.1093/bioinformatics/bti476. 15890747

[ref7] Bertinetto C.; Engel J.; Jansen J. ANOVA simultaneous component analysis: A tutorial review. Anal. Chim. Acta X 2020, 6, 100061 10.1016/j.acax.2020.100061. 33392497 PMC7772684

[ref8] Di Donato F.; Biancolillo A.; Ferretti A.; D’Archivio A. A.; Marini F. Near infrared spectroscopy coupled to chemometrics for the authentication of donkey milk. J. Food Compos. Anal. 2023, 115, 105017 10.1016/j.jfca.2022.105017.

[ref9] Cheng W.; Sørensen K. M.; Mongi R. J.; Ndabikunze B. K.; Chove B. E.; Sun D. W.; Engelsen S. B. A comparative study of mango solar drying methods by visible and near-infrared spectroscopy coupled with ANOVA-simultaneous component analysis (ASCA). LWT 2019, 112, 108214 10.1016/j.lwt.2019.05.112.

[ref10] Amigo J. M.; Del Olmo A.; Engelsen M. M.; Lundkvist H.; Engelsen S. B. Staling of white wheat bread crumb and effect of maltogenic α-amylases. Part 2: Monitoring the staling process by using near infrared spectroscopy and chemometrics. Food Chem. 2019, 297, 124946 10.1016/j.foodchem.2019.06.013. 31253319

[ref11] Bevilacqua M.; Bucci R.; Materazzi S.; Marini F. Application of near infrared (NIR) spectroscopy coupled to chemometrics for dried egg-pasta characterization and egg content quantification. Food Chem. 2013, 140 (4), 726–734. 10.1016/j.foodchem.2012.11.018. 23692759

[ref12] Rust A.; Marini F.; Allsopp M.; Williams P. J.; Manley M. Application of ANOVA-simultaneous component analysis to quantify and characterise effects of age, temperature, syrup adulteration and irradiation on near-infrared (NIR) spectral data of honey. Spectrochim. Acta A Mol. Biomol. Spectrosc. 2021, 253, 119546 10.1016/j.saa.2021.119546. 33677373

[ref13] van Rooyen J.; Marini F.; Orth S. H.; Oyeyinka S. A.; Simsek S.; Manley M. Effect of wheat roasting conditions and wheat type on short-wave infrared (SWIR) spectral data of whole and milled wheat by ANOVA-simultaneous component analysis. Spectrochimica Acta Part A: Molecular and Biomolecular Spectroscopy 2023, 303, 123160 10.1016/j.saa.2023.123160. 37481843

[ref14] Thiel M.; Feraud B.; Govaerts B. ASCA+ and APCA+: Extensions of ASCA and APCA in the analysis of unbalanced multifactorial designs. J. Chemom. 2017, 31 (6), e2895 10.1002/cem.2895.

[ref15] Morais C. L.; Lima K. M.; Singh M.; Martin F. L. Tutorial: multivariate classification for vibrational spectroscopy in biological samples. Nat. Protoc. 2020, 15 (7), 2143–2162. 10.1038/s41596-020-0322-8. 32555465

[ref16] Agelet L. E.; Hurburgh C. R. Jr A tutorial on near infrared spectroscopy and its calibration. Crit. Rev. Anal. Chem. 2010, 40 (4), 246–260. 10.1080/10408347.2010.515468.

[ref17] Geladi P.; MacDougall D.; Martens H. Linearization and scatter-correction for near-infrared reflectance spectra of meat. Appl. Spectrosc. 1985, 39 (3), 491–500. 10.1366/0003702854248656.

[ref18] Barnes R.; Dhanoa M. S.; Lister S. J. Standard normal variate transformation and de-trending of near-infrared diffuse reflectance spectra. Appl. Spectrosc. 1989, 43 (5), 772–777. 10.1366/0003702894202201.

[ref19] Martens H.; Nielsen J. P.; Engelsen S. B. Light scattering and light absorbance separated by extended multiplicative signal correction. Application to near-infrared transmission analysis of powder mixtures. Anal. Chem. 2003, 75 (3), 394–404. 10.1021/ac020194w. 12585463

[ref20] AGES. Österreichische Beschreibende Sortenliste 2023 Landwirtschaftliche Pflanzenarten. 21/2023, ISSN 1560–635X; Republik Österreich, 2023.

[ref21] Hiebl J.; Frei C. Daily precipitation grids for Austria since 1961—Development and evaluation of a spatial dataset for hydroclimatic monitoring and modelling. Theor. Appl. Climatol. 2018, 132, 327–345. 10.1007/s00704-017-2093-x.

[ref22] Pena-Pereira F.; Wojnowski W.; Tobiszewski M. AGREE—Analytical GREEnness Metric Approach and Software. Anal. Chem. 2020, 92 (14), 10076–10082. 10.1021/acs.analchem.0c01887. 32538619 PMC7588019

[ref23] Stevens A.; Ramirez-Lopez L. An introduction to the prospectr package. 2022. https://antoinestevens.github.io/prospectr/ (accessed 10.09.2024).

[ref24] Liland K. H. EMSC: Extended Multiplicative Signal Correction. 2021. https://CRAN.R-project.org/package=EMSC (accessed 10.09.2024).

[ref25] Kucheryavskiy S. mdatools–R package for chemometrics. Chemom. Intell. Lab. Syst. 2020, 198, 103937 10.1016/j.chemolab.2020.103937.

[ref26] Thiel M.; Benaiche N.; Martin M.; Franceschini S.; Van Oirbeek R.; Govaerts B. limpca: An R package for the linear modeling of high-dimensional designed data based on ASCA/APCA family of methods. J. Chemom. 2023, 37 (7), e3482 10.1002/cem.3482.

[ref27] Wickham H. ggplot2 - Elegant Graphics for Data Analysis; Springer, 2016.

[ref28] Saveliev M.; Panchuk V.; Kirsanov D. Math is greener than chemistry: Assessing green chemistry impact of chemometrics. Trends Anal. Chem. 2024, 172, 117556 10.1016/j.trac.2024.117556.

[ref29] De Girolamo A.; Cortese M.; Cervellieri S.; Lippolis V.; Pascale M.; Logrieco A. F.; Suman M. Tracing the geographical origin of durum wheat by FT-NIR spectroscopy. Foods 2019, 8 (10), 450 10.3390/foods8100450. 31581610 PMC6835725

[ref30] Wadood S. A.; Guo B.; Zhang X.; Wei Y. Geographical origin discrimination of wheat kernel and white flour using near-infrared reflectance spectroscopy fingerprinting coupled with chemometrics. Int. J. Food Sci. Technol. 2019, 54 (6), 2045–2054. 10.1111/ijfs.14105.

[ref31] Zhao H.; Guo B.; Wei Y.; Zhang B. Near infrared reflectance spectroscopy for determination of the geographical origin of wheat. Food Chem. 2013, 138 (2–3), 1902–1907. 10.1016/j.foodchem.2012.11.037. 23411323

[ref32] Zhao H.; Guo B.; Wei Y.; Zhang B. Effects of grown origin, genotype, harvest year, and their interactions of wheat kernels on near infrared spectral fingerprints for geographical traceability. Food Chem. 2014, 152, 316–322. 10.1016/j.foodchem.2013.11.122. 24444943

[ref33] González-Martín M. I.; Moncada G. W.; González-Pérez C.; San Martín N. Z.; López-González F.; Ortega I. L.; Hernández-Hierro J.-M. Chilean flour and wheat grain: Tracing their origin using near infrared spectroscopy and chemometrics. Food Chem. 2014, 145, 802–806. 10.1016/j.foodchem.2013.08.103. 24128548

[ref34] Gorla G.; Taborelli P.; Ahmed H. J.; Alamprese C.; Grassi S.; Boqué R.; Riu J.; Giussani B. Miniaturized NIR spectrometers in a nutshell: Shining light over sources of variance. Chemosensors 2023, 11 (3), 182 10.3390/chemosensors11030182.

[ref35] Gorla G.; Taborelli P.; Giussani B. A Multivariate Analysis-Driven Workflow to Tackle Uncertainties in Miniaturized NIR Data. Molecules 2023, 28 (24), 7999 10.3390/molecules28247999. 38138488 PMC10745448

