# Supplementary material for: Simplifying Wheat Quality Assessment: Using Near-Infrared Spectroscopy and Analysis of Variance Simultaneous Component Analysis to Study Regional and Annual Effects
Source: ACS Meas Sci Au. 2024 Oct 4;4(6):695–701. doi: 10.1021/acsmeasuresciau.4c00044 (PMC11659997; doi:10.1021/acsmeasuresciau.4c00044)
Supplement: Supplementary file 2 — tg4c00044_si_002.pdf [file tg4c00044_si_002.pdf]

Supporting Information

Simplifying Wheat Quality Assessment: Using Near-Infrared Spectroscopy and Analysis of Variance Simultaneous Component Analysis to Study Regional and Annual Effects

Stephan Freitag, [1*] Maximilian Anlanger,[1] Maximilian Lippl,[2] Klemens Mechtler,[3] Elisabeth Reiter,[2] Heinrich Grausgruber,[4] Rudolf Krska [1,5,6]

1 BOKU University, Department of Agrobiotechnology, IFA-Tulln, Institute of Bioanalytics and Agro-Metabolomics, Konrad-Lorenz-Str. 20, 3430 Tulln an der Donau, Austria

2 Austrian Agency for Health and Food Safety GmbH, Institute for Animal Nutrition and Feed, Spargelfeldstr. 192, 1220 Vienna, Austria

3 Austrian Agency for Health and Food Safety GmbH, Institute for Sustainable Plant Production, Spargelfeldstr. 192, 1220 Vienna, Austria

4 BOKU University, Department of Crop Sciences, Institute of Plant Breeding, Konrad-Lorenz-Str. 24, 3430 Tulln an der Donau, Austria

5 FFoQSI GmbH – Austrian Competence Centre for Feed and Food Quality, Safety and Innovation, Technopark 1C, 3430 Tulln an der Donau, Austria

6 Institute for Global Food Security, School of Biological Sciences, Queens University Belfast, University Road, Belfast, BT7 1NN, Northern Ireland, United Kingdom

InSupporting Table 1 The GPS locations of the sample sites are found.

Supporting Table 1: GPS data of the sampling sites

Longitude	Latitude	Location name
13.89546	48.05050	Bad Wimsbach
15.53271	48.24277	Flinsbach
13.37209	48.32621	Reichersberg
15.23972	48.13526	Zinsenhof

In Supporting Table 2 results of analysis of variance simualtenious component analysis (ASCA) found decomposition are found.

Supporting Table 2: Amount of variance attributed to certain effects after using analysis of variance simultaneous component analysis paired with different spectra processing methods. SNV = standard normal variate; MSC = multiplicative signal correction; EMSC = extended multiplicative signal correction

Pre-processing method	Year	Region	Year:Region	Residuals
none	48.2	7.2	4.5	38.5
SNV	53.9	14.1	9.6	21.4
MSC	53.9	14.1	9.6	21.4
EMSC	60.7	14.8	6.8	17.0
First derivative	48.8	11.3	6.1	32.4
Second derivative	46.2	10.2	7.9	34.4

In Supporting Table 3 & 4 results of ASCA are found for decomposition and dimesion reduction along the effects of the year and sample site when using different spectra-preprocessing strategies.

Supporting Table 3: Influence of spectra pre-processing analysis of variance simultaneous component analysis decomposition for dimension reduction for the effect of the year.

Pre-processing method	Variance PC1 [%]	Variance PC2 [%]
none	97.6	2.4
SNV	57.5	42.5
MSC	57.5	42.5
EMSC	73.6	26.4
First derivative	77.9	22.1
Second derivative	77.1	22.9

Supporting Table 4: Influence of spectra pre-processing analysis of variance simultaneous component analysis decomposition for dimension reduction for the effect of the year.

Pre-processing method	Variance PC1 [%]	Variance PC2 [%]
none	97.8	1.5
SNV	87.2	12.2
MSC	87.2	12.2
EMSC	94.9	4.3
First derivative	89.4	9.3
Second derivative	86.0	11.6

In Supporting Figure 2-6 score and loading plots obtained by ASCA for the effect of the year and region using different spectra pre-processing strategies are presented.

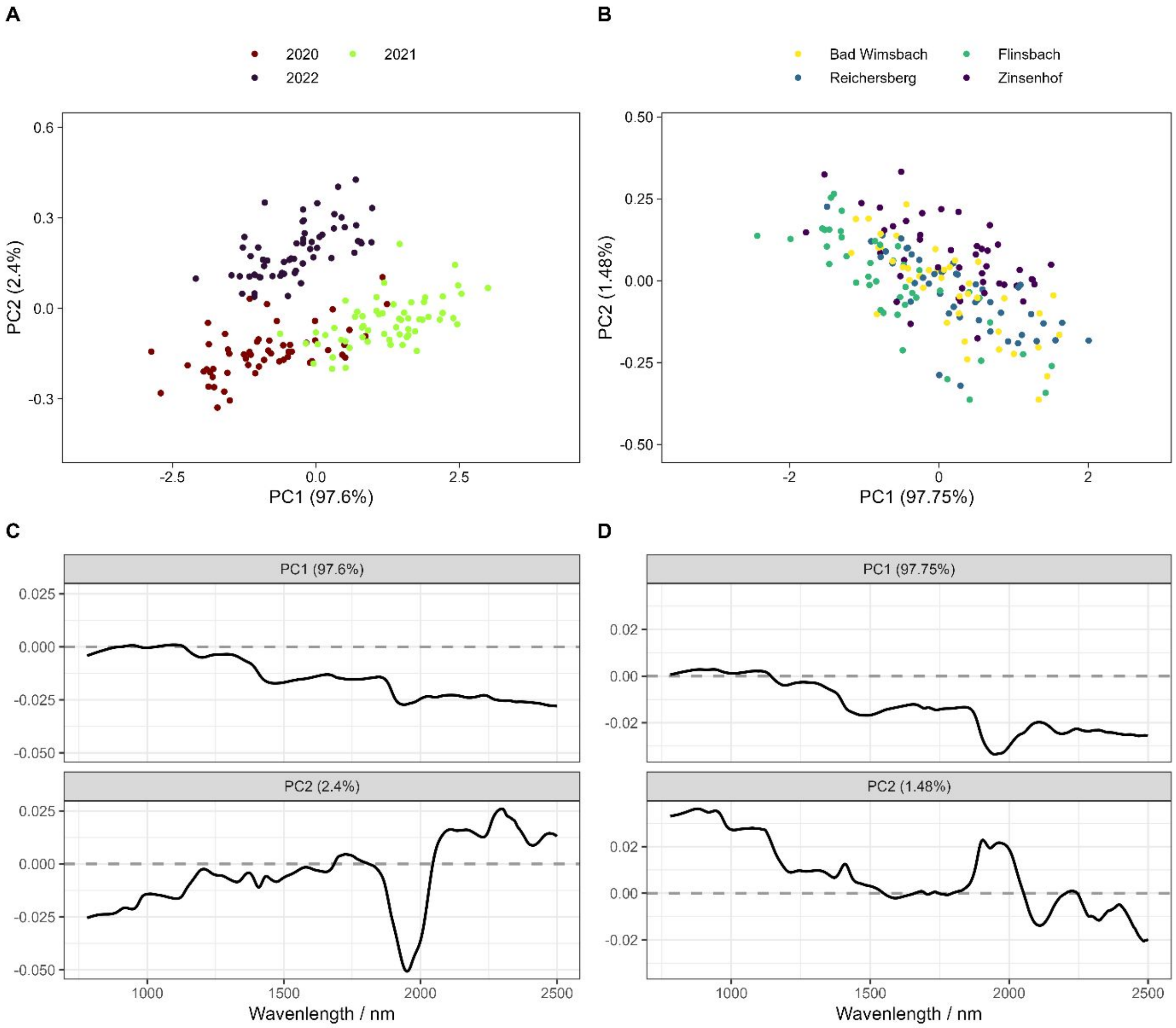

Supporting Figure 1: Score and loadings plots obtained by analysis of variance simultaneous component analysis (ASCA) using unprocessed near-infrared spectra. ASCA score plots for the effect of the year (A) or region (B) as well as the corresponding loading plots for the effect of the year (C) or region (D).

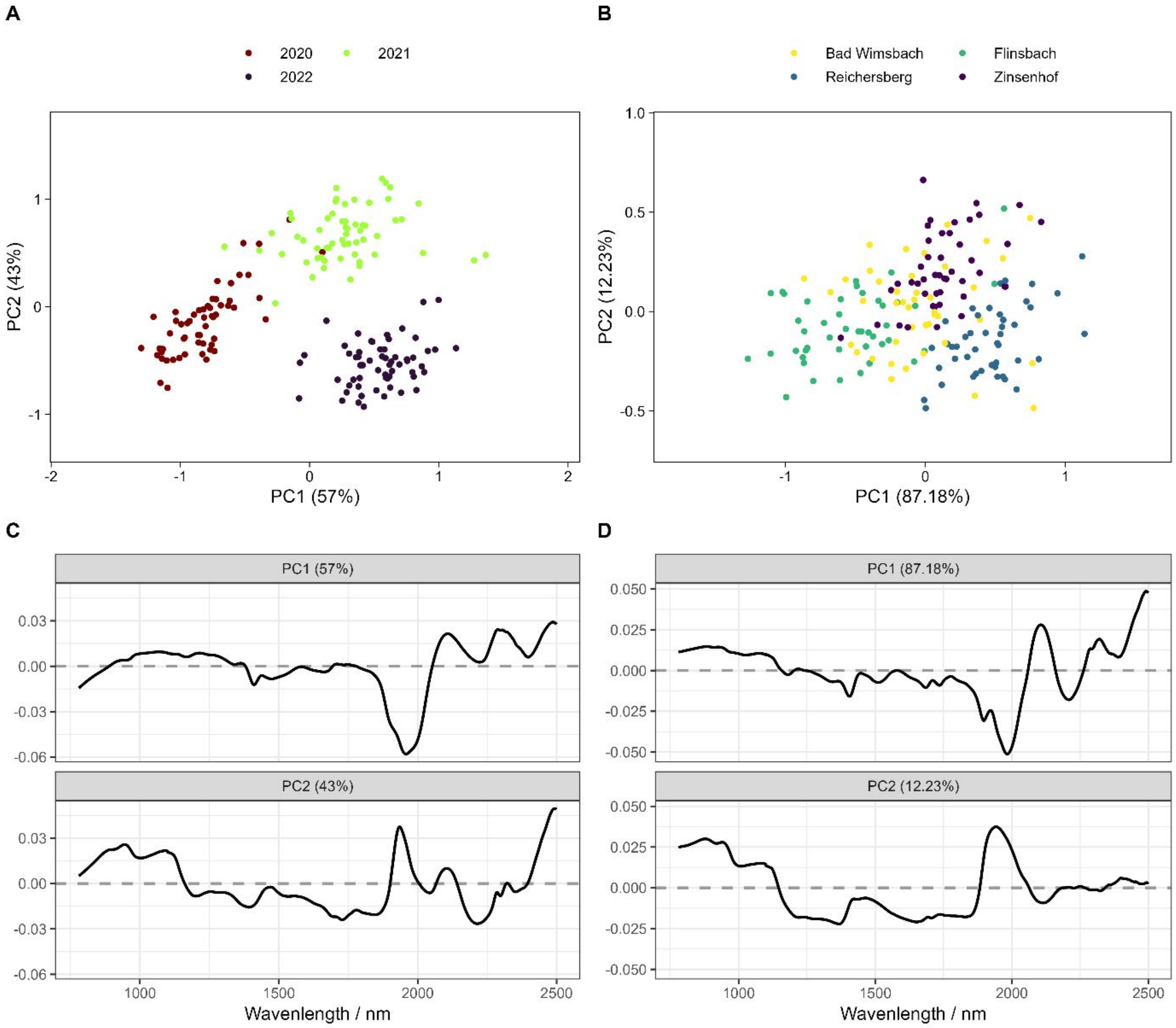

Supporting Figure 2: Score and loadings plots obtained by analysis of variance simultaneous component analysis (ASCA) using multi (SNV) corrected near-infrared spectra. ASCA score plots for the effect of the year (A) or region (B) as well as the corresponding loading plots for the effect of the year (C) or region (D).

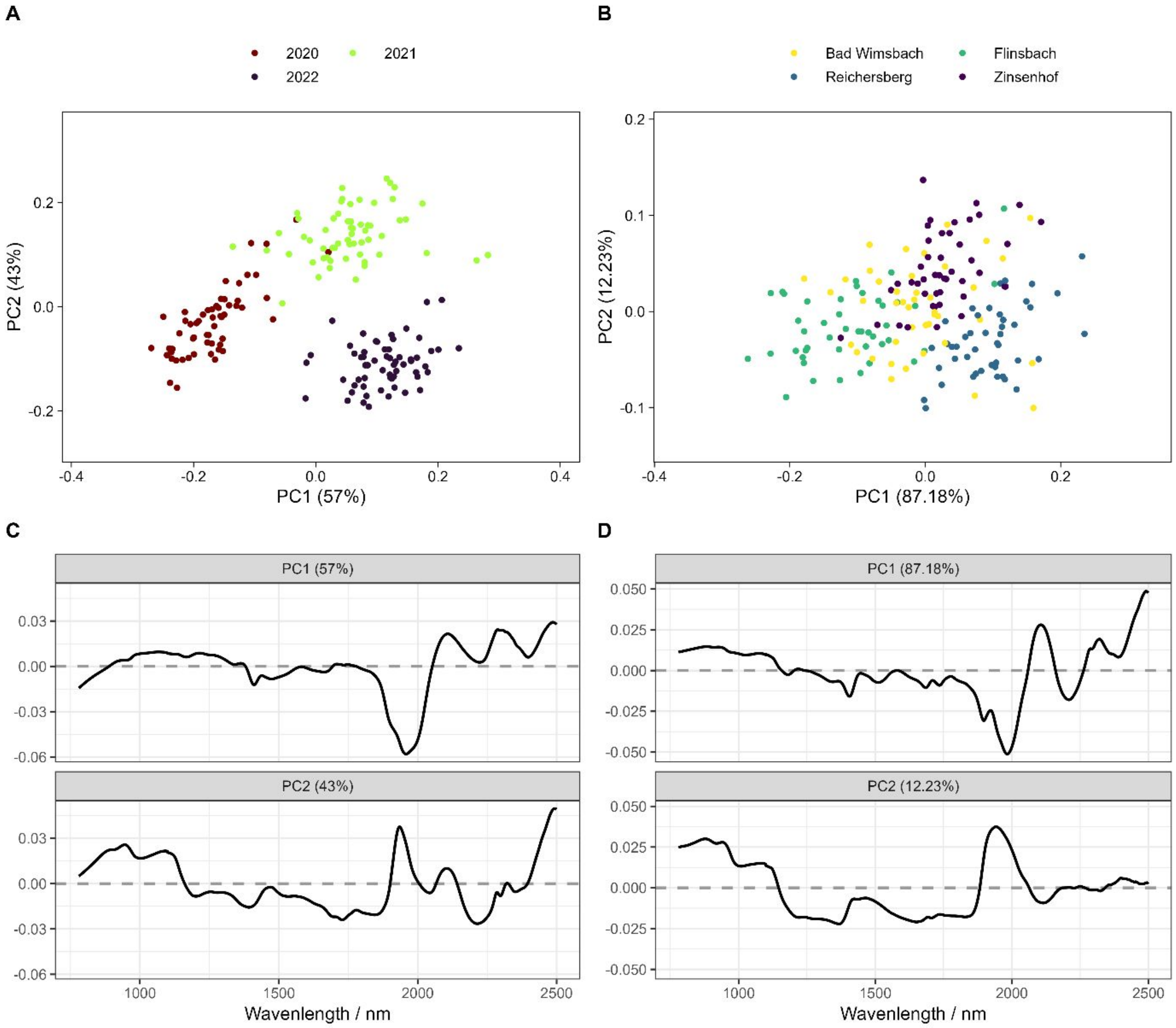

Supporting Figure 3: Score and loadings plots obtained by analysis of variance simultaneous component analysis (ASCA) using multiplicative signal corrected (MSC) near-infrared spectra. ASCA score plots for the effect of the year (A) or region (B) as well as the corresponding loading plots for the effect of the year (C) or region (D).

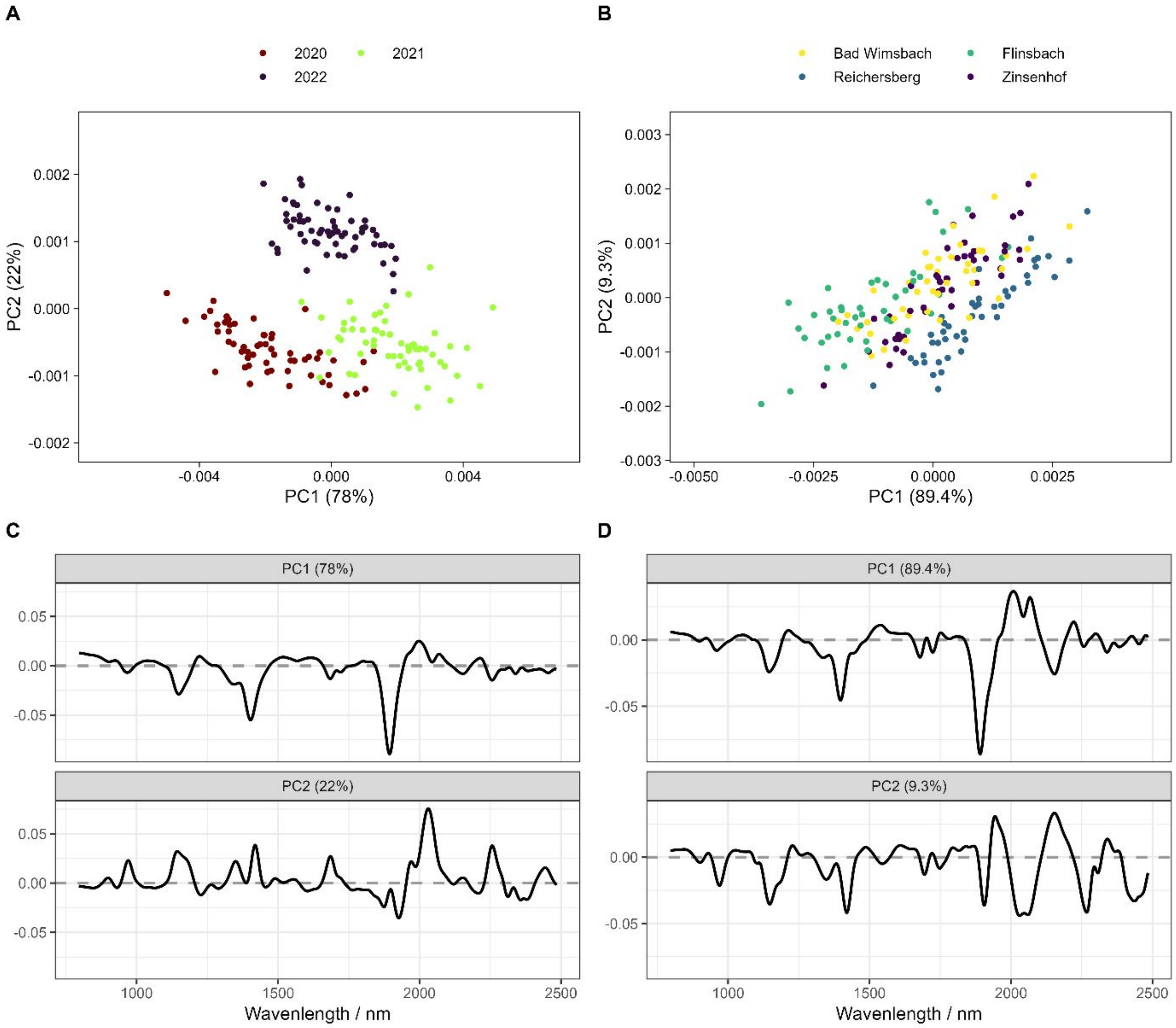

Supporting Figure 4: Score and loadings plots obtained by analysis of variance simultaneous component analysis (ASCA) using the first derivative of the near-infrared spectra. ASCA score plots for the effect of the year (A) or region (B) as well as the corresponding loading plots for the effect of the year (C) or region (D).

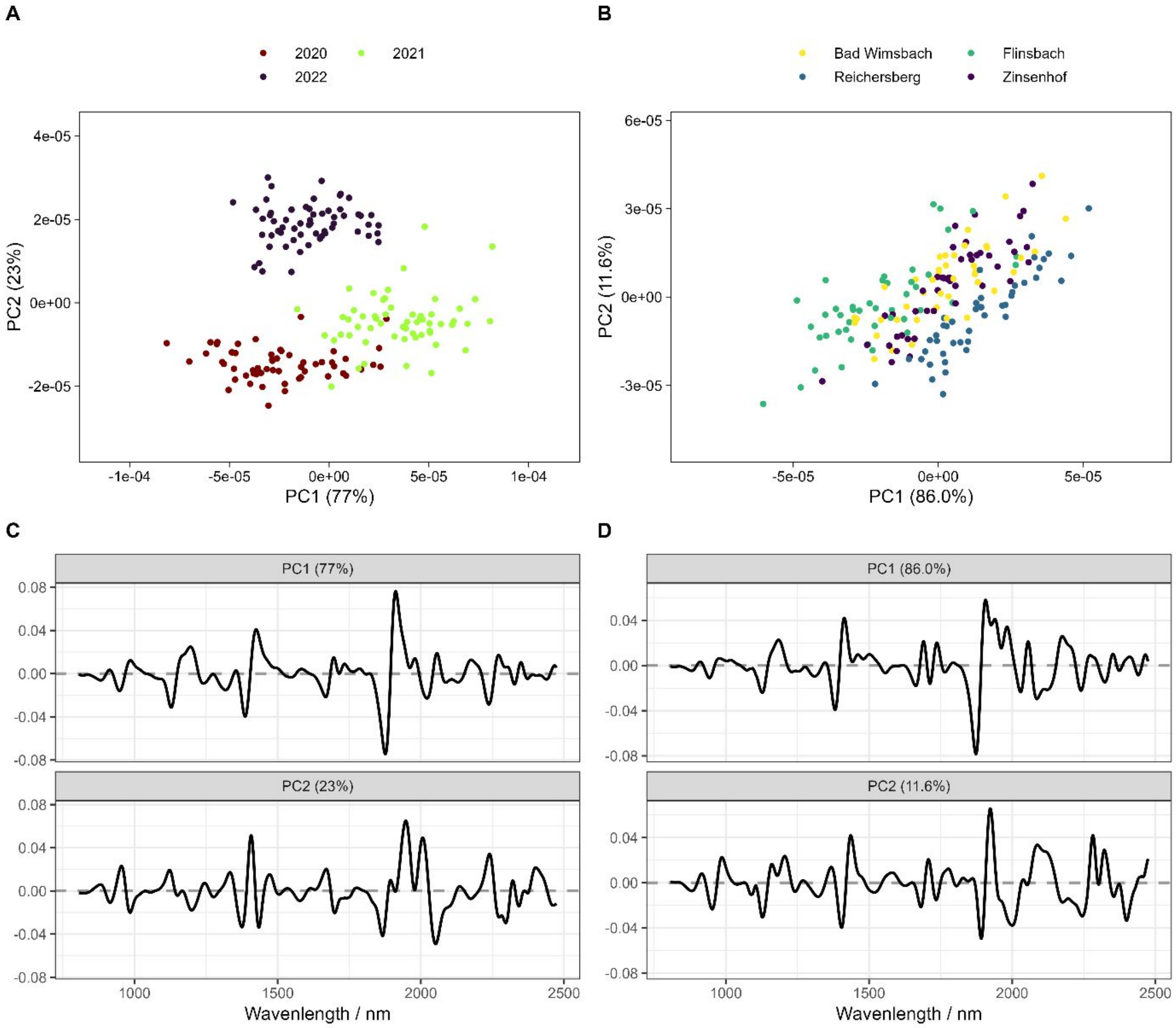

Supporting Figure 5: Score and loadings plots obtained by analysis of variance simultaneous component analysis (ASCA) using the second derivative of the near-infrared spectra. ASCA score plots for the effect of the year (A) or region (B) as well as the corresponding loading plots for the effect of the year (C) or region (D).
